# Long-Term Outcomes of Perinatal Hypoxia and Asphyxia at an Early School Age

**DOI:** 10.3390/medicina57090988

**Published:** 2021-09-18

**Authors:** Renata Dzikienė, Saulius Lukoševičius, Jūratė Laurynaitienė, Vitalija Marmienė, Irena Nedzelskienė, Rasa Tamelienė, Inesa Rimdeikienė, Aušrelė Kudrevičienė

**Affiliations:** 1 Department of Neonatology, Lithuanian University of Health Sciences, LT-50009 Kaunas, Lithuania; Rasa.Tameliene@kaunoklinikos.lt (R.T.); Ausrele.Kudreviciene@lsmuni.lt (A.K.); 2 Department of Radiology, Lithuanian University of Health Sciences, LT-50009 Kaunas, Lithuania; Saulius.Lukosevicius@kaunoklinikos.lt; 3 Department of Neurology, Lithuanian University of Health Sciences, LT-50009 Kaunas, Lithuania; Jurate.Laurynaitiene@kaunoklinikos.lt; 4 Department of Psychiatry, Lithuanian University of Health Sciences, LT-50009 Kaunas, Lithuania; vitalija@gmail.com; 5 Department of Dental and Oral Disease, Lithuanian University of Health Sciences, LT-50009 Kaunas, Lithuania; irena.nedzelskiene@lsmuni.lt; 6 Department of Rehabilitation, Lithuanian University of Health Sciences, LT-50009 Kaunas, Lithuania; inesa.rimdeikiene@kaunoklinikos.lt

**Keywords:** perinatal asphyxia, long-term outcomes, early school age, WISC-III, neonatal hypoxic-ischemic encephalopathy

## Abstract

*Background and Objectives:* Late long-term outcomes of perinatal asphyxia (PA) in school-age are often unclear. To assess long-term outcomes at an early school age in children who had experienced perinatal hypoxia or asphyxia, where therapeutic hypothermia was not applied. *Materials and Methods*: The case group children were 8–9-year-old children (*n* = 32) who were born at full term and experienced hypoxia or asphyxia at birth, where therapeutic hypothermia (TH) was not applied. The control group consisted of 8–9-year-old children (*n* = 16) born without hypoxia. A structured neurological examination was performed at an early school age. The neuromotor function was assessed using the Gross Motor Function Classification System (GMFCS). Health-related quality-of-life was assessed using the Health Utilities Index (HUI) questionnaire. Intellectual abilities were assessed using the Wechsler Intelligence Scale for Children (WISC). *Results:* The case group, compared with controls, had significantly (*p* = 0.002) lower mean [SD] full-scale IQ (87(16.86) vs. 107(12.15)), verbal-scale IQ (89(17.45) vs. 105(11.55)), verbal comprehension index (89(17.36) vs. 105(10.74)), working memory index (89(15.68) vs. 104(11.84)), performance IQ (87(16.51) vs. 108(15.48)) and perceptual organization index (85(15.71) vs. 105(15.93)). We did not find any significant differences in the incidence of disorders of neurological examination, movement abilities and health-related quality of life at an early school age between the case and the control group children. *Conclusion:* In children who experienced perinatal asphyxia but did not have cerebral paralysis (CP), where therapeutic hypothermia was not applied, cognitive assessment scores at an early school age were significantly lower compared to those in the group of healthy children, and were at a low average level.

## 1. Introduction

Perinatal asphyxia in full-term neonates is one of the most common causes of neonatal morbidity and mortality in most countries of the world. According to the literature data, 20 out of 1000 live births require extensive resuscitation, and clinical and biochemical signs of perinatal asphyxia subsequently emerge. About 15–20% of such neonates die during the neonatal period, up to 25% of the survivors are diagnosed with neurological disorders, and, in 10 to 30% of such neonates, the psychomotor development slows down [1]. Long-term outcomes of perinatal asphyxia include severe disability (impaired mental and motor development, cortical blindness, sensorineural hearing loss, epilepsy, and cerebral palsy) and death.

A number of studies analyzing the long-term outcomes of perinatal asphyxia at a preschool age showed that the incidence of severe disability ranged from 11 to 19% [2,3], the incidence of cerebral palsy from 5.5 to 52% [3,4,5,6,7,8], the incidence of various motor disorders from 1.3 to 40% [4,7,9,10,11], the incidence of hearing impairment from 2 to 20% [3,7,10], the incidence of visual impairment from 1.8 to 40% [3,6,7,12], and the incidence of speech disorders from 4.2 to 21% [3,4,5]. According to the literature data, children who required post-natal resuscitation and experienced neonatal hypoxic-ischemic encephalopathy (HIE) develop cognitive and behavioral disorders at school age, but the evidence for this is scarce [13,14]. There are insufficient data on which psychomotor disorders children who experienced perinatal asphyxia or hypoxia and did not undergo therapeutic hypothermia have at an early school age, and what problems they face when learning.

The aim of our study was to evaluate long-term outcomes at an early school age in children who experienced perinatal hypoxia or asphyxia.

## 2. Materials and Methods

**Figure 1:**
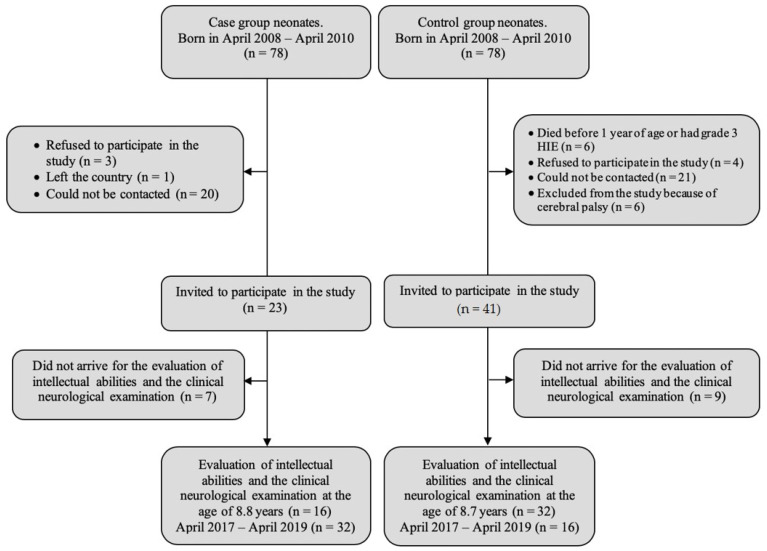
The structure of the study. HIE—hypoxic-ischemic encephalopathy.

This study was approved by the decision of Kaunas Regional Biomedical Research Ethics Committee passed at the Committee session on 4 April 2017 (protocol No. BE-2-13). The representatives of all children (mothers and/or fathers) gave written consent to participate in the study after they were familiarized with its aim and methods. A prospective case control study was performed at the Clinical Department of Neonatology of the Hospital of the Lithuanian University of Health Sciences (LSMU) between April 2008 and April 2019. The study group consisted of 32 children of an early school age (8–9 years) who were born at full term (≥37 weeks of gestation), experienced hypoxia or asphyxia during birth, and were treated at the LSMU Perinatal Center between April 2008 and April 2010. Therapeutic hypothermia was not applied on the patients, because we began using it a few years later in Lithuania. The control group consisted of 16 children of an early school age (8–9 years old) who were born at the Clinic of Obstetrics and Gynecology of the LSMU Hospital between April 2008 and April 2010, did not have hypoxia, and left the hospital healthy (Figure 1). The children’ characteristics are presented in Table 1.

In 2007, the WHO presented the criteria for hypoxia/asphyxia at birth. The inclusion criteria were selected on the basis of this [15]. Inclusion criteria for the case group children were as follows: full-term (≥37 weeks of gestation) neonates born, required resuscitation, Apgar score at 5 min after birth ≤7 points, fetal acidosis (umbilical artery blood pH < 7.2) or neonatal acidosis (capillary blood pH within the first hour after birth <7.3). Parental agreement was required for the child’s participation in the study.

The exclusion criteria for the case group neonates were the following: full-term (≥37 weeks of gestation) neonates with congenital developmental or chromosome abnormalities, hemolytic disease of the newborn, congenital brain infection or severe sepsis with hemodynamic disturbances, or suspected metabolic diseases.

Inclusion criteria for the control group children were as follows: full-term (≥37 weeks of gestation) neonates who did not require resuscitation, Apgar score on the 1st and the 5th minute of life ≥8 points, and no neonatal pathologies. Parental agreement was required for the child’s participation in the study.

### 2.1. Clinical Evaluation of Neurological Condition during the Neonatal Period

After the resuscitation, the neurological condition was evaluated on a daily basis for the first three days of life. The HIE grade was evaluated using the modified Sarnat and Sarnat scale [16].

### 2.2. Mental Development at the Age of 1 Year

Mental development was evaluated in children aged 12 ± 1 months using the mental scale of the Bayley Scale for Infant Development, 2nd ed. (BSID-II) [17]. Mental development was considered to be normal when the mental development index was >85, slight mental retardation was diagnosed when the mental development index was 70–85 (−1 SD), and marked mental retardation was diagnosed when the mental development index was <70 (−2 SD).

### 2.3. Assessment of Intellectual Abilities at an Early School Age

The intellectual abilities of the case and the control group children were assessed using the Wechsler Intelligence Scale for Children, validated in Lithuania (WISC-IIILT). The intellectual abilities were assessed by calculating the full, verbal, and non-verbal intelligence quotient (IQ) score. Qualitative data interpretation was applied for the evaluation of the children’s abilities (Table 2) [18]. All the children were evaluated by a single psychologist who did not know to which group the children belonged.

### 2.4. Clinical Neurological Examination and Evaluation of the Motor Function at an Early School Age

A structured neurological examination was performed, including the assessment of cranial nerves, upper and lower extremities, cerebellar function, gait, and muscle tone, as well as neurological inspection. The children’s neuromotor function was assessed at an early school age (8–9 years), using the Gross Motor Function Classification System. The scores on the two assessments ranged from 1 to 5 points, with a higher score meaning a more severe impairment [19]. The scoring on the Gross Motor Function Classification System was as follows: Level 1—Capable of walking independently but may have gait problems; Level 2—Able to walk in most settings, but has, at best, only minimal ability to perform gross motor skills such as running and jumping; Level 3—Child walks using a hand-held mobility device in most indoor settings. When seated, the child may require a seatbelt for pelvic alignment and balance. When traveling long distances, the child uses some form of wheeled mobility; Level 4—Child uses methods of mobility that require physical assistance or powered mobility in most settings. At school, outdoors, and in the community, the child is transported in a manual wheelchair or uses powered mobility; Level 5—Child is transported in a manual wheelchair in all settings. The child is limited in his or her ability to maintain antigravity head and trunk postures and to control arm and leg movements. All the children were evaluated by a single neurologist who did not know to which group the children belonged.

### 2.5. Evaluation of Health-Related Quality of Life (HRQL) at an Early School Age

Parents completed the Health Utilities Index questionnaire on behalf of their children [20]. The HUI questionnaire helps to assess the overall health status. It has two scoring systems: HUI2 and HUI3. The HUI3 questionnaire contains questions about eight attributes: vision, hearing, speech, ambulation, dexterity, emotion, cognition, and pain. The HUI2 questionnaire contains questions about six attributes: sensation, mobility, emotion, cognition, self-care, and pain. As the HUI3 questionnaire contains a more detailed description of the general health status, the HUI3 was used as the preferred measure in this study. The HUI2 was used for further analysis.

For each question in the HUI3 questionnaire, the respondents chose one of the provided descriptions, covering different levels of abilities—from the best or normal ability (level 1) to the most severe disability (level 2, 3, 4, 5, or 6, depending on the attribute and the scoring system). For example, there are six questions for the assessment of vision, describing the level of visual ability from “Able to see well enough to read ordinary newsprint and recognize a friend on the other side of the street, without glasses or contact lenses” (level 1) to “Unable to see at all” (level 6).

### 2.6. Statistical Methods of Data Analysis

Statistical analysis of the data was performed using software packages SPSS 22.0 (SPSS Inc., Chicago, IL, USA) for data storage and analysis. All parametric data are expressed as means and standard deviations. The Kolmogorov-Smirnov test was used for the evaluation of the distribution of quantitative data. When the distribution of the variables was normal, Student’s t test was used to compare quantitative sizes of two independent samples. The Mann-Whitney U test was used to compare non-normally distributed variables. One-way analysis of variance (ANOVA) was used to compare more than two independent groups. The Least Significant Difference (Bonferroni) post hoc test was used for multiple paired comparisons. The Kruskal–Wallis test was used to compare non-normally distributed variables. The interdependence of qualitative evidence was evaluated by the chi-square (χ2) test. Depending on the sample size, exact (for a small size) or asymptomatic criteria were used. Differences between the groups were considered significant when the level of significance was *p* < 0.05.

## 3. Results

We did not find any significant differences in the incidence of disorders of neurological examination, motor function and health-related quality-of-life at an early school age between the case and the control group children (Table 3).

In the case group, the results of cognitive assessment at an early school age were significantly lower (*p* < 0.05) and were at a low average level (Table 4), but there was no significant difference in the frequency of IQ scores of 85 or lower between the case and the control groups (Table 5).

In 12.9% (*n* = 4) of the newborns in the case group, a slight retardation of mental development was found at the age of 1 year. At an early school age, the full IQ, verbal IQ, verbal comprehension index and working memory index scores of these children were within the borderline range and differed significantly from the IQ scores of children whose mental development was normal at the age of 1 year (*p* < 0.2) (Table 6).

**Figure 2:**
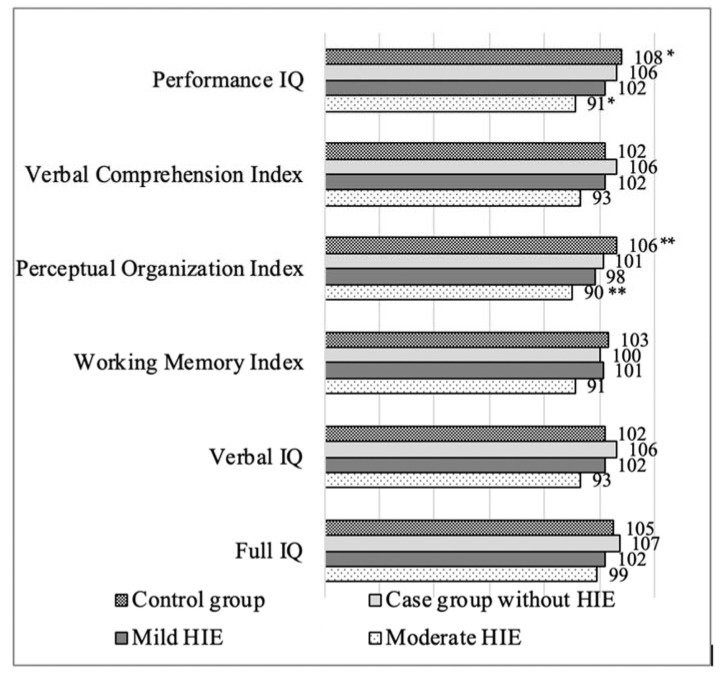
Mean IQ coefficient scores at an early school age in children with respect to HIE grade. The text continues here. IQ—intelligence quotient; HIE—hypoxic ischemic encephalopathy; * *p* = 0.02; ** *p* = 0.021.

We analyzed long-term outcomes depending on the HIE, according to Sarnat and Sarnat. The assessment of intellectual abilities at an early school age showed that, in patients who experienced moderate HIE, Performance IQ and Perceptual Organization Index scores at an early school age were significantly lower (*p* = 0.02; *p* = 0.021) than those in the control group children (Figure 2). Children with HIE had a statistically higher incidence of epilepsy: in the control group 0%, mild HIE—7.1%, moderate HIE—25% (*p* = 0.026). No other statistically significant neurological changes dependent on the stage of HIE were observed.

## 4. Discussion

During the neonatal period, when monitoring the condition of a newborn that has experienced perinatal hypoxia or asphyxia, we usually try to predict poor early outcomes—severe motor and mental developmental disorders. However, it is difficult to predict what health problems those who are not diagnosed with cerebral palsy might develop at an early school age. We analyzed long-term outcomes at an early school age in children who were born at full term, experienced hypoxia or asphyxia at birth, and did not undergo TH. The evaluation of the clinical neurological condition showed that 43.8% of the children in the case group had minor abnormal signs, and 12.5% of them had epilepsy, but we did not find any significant differences in the incidence of such disorders between the case and the control group children.

When assessing neurological function in healthy children and children who experienced PA and underwent TH, Campbell et al. found that there were significantly more neurological disorders in the case group children [21]. The results of a study by Liu X. et al. also showed that 13% of the children who experienced PA were diagnosed with epilepsy at 4–8 years of age [22].

The results of a study in Switzerland also showed that children with HIE who did not have any major disability were at an increased risk for long-term motor deficits [23]. When assessing the motor function, we found mild motor function (levels I and II) in 34.0% of the case group children, but we did not find any significant differences in the frequency of such disorders between the case and control group children. Azzopardi et al. found level I-II abnormalities in 22% of the children who experienced PA, and 41% of such children had level III-IV abnormalities [24]. However, this study also included children who were diagnosed with CP, while such children were excluded from our study.

The assessment of health-related quality-of-life showed that 12.5% of the case group children had mild vision disorders, 3.1% had hearing disorders, 6.3% had speech disorders, 9.4% experienced pain, 25.0% of the children had ambulation disorders, 6.2% had dexterity disorders, and 31.3% had cognitive disorders, but we found no significant differences in the incidence of such disorders between the case and the control group children. Campbell et al. analyzed long-term outcomes at an early school age in children who had experienced PA. They used the Health Utilities Index (HUI) questionnaire in their study. The results of their study showed that, at the age of 6–7 years, the incidence of hearing disorders among such children was almost twice as high (5.97%) as that among children who had not experienced PA, the incidence of vision disorders was slightly higher (18.8%), and the incidence of speech disorders was almost seven times higher (41.4%) [21]. Children in this study did not have cerebral palsy, but had undergone TH. Azzopardi et al. analyzed the effects of hypothermia after perinatal asphyxia and found that children with perinatal asphyxia who had not undergone TH had a similar incidence of vision impairment (12%), compared to children who had undergone TH, while the incidence of hearing disorders was almost three times higher (11%) [24].

Our study showed that, in children who experienced PA, cognitive assessment scores at an early school age were significantly lower (*p* < 0.005) than those in the control group children’s, and were at the low average level. Lee-Kelland et al. evaluated long-term outcomes in children aged 6–8 years who had experienced PA and undergone TH. These authors also found that cognitive assessment scores in these children were significantly lower than in healthy children [25]. Similarly, to our findings, other researchers [25,26] also found that, in children who had experienced PA, the full IQ score at an early school age fluctuated at the borderline and low average levels, verbal IQ, performance IQ and the Working Memory Index fluctuated at the low average and average levels, and the Perceptual Organization Index fluctuated at the low average level.

According to studies performed by Azzopardi et al. and Campbell et al., 36–37% of children who experienced PA had a full IQ score of ≤ 85 [21,24]. Our study showed that 18.8% of the children who experienced PA had a full IQ score of ≤ 85, but there was no significant difference in the full IQ score between the case and the control group children. However, for children who experienced moderate HIE, performance IQ and Perceptual Organization Index scores at an early school age were significantly lower (*p* = 0.02) than those in the control group children.

In patients who experience asphyxia/hypoxia at birth, long-term outcomes are not always clear during the first year of life. The results of a number of studies suggest that normal outcomes of perinatal hypoxia or asphyxia during infancy do not imply that the outcomes are going to be good at a school age [22,23,27,28,29]. At the age of one year, 12.9% of the children were found to have mild mental retardation. However, at an early school age, 18.8% of children had a full IQ score of 85 or below, 28.1% of children had performance IQ and verbal IQ scores of 85 or below, and 18.8% of the study group children had a verbal IQ score of 85 or below. All children who were found to have mild mental retardation at one year of age demonstrated IQ scores within the borderline range at the early school age, which differed significantly from the IQ scores of children whose mental development at the age of 1 year was normal.

Our study has several limitations. One of the limitations of our study is a small sample size in the case and the control groups. When long-term outcomes at school age were assessed, a large proportion of children were lost for a variety of reasons. We were unable to evaluate a large proportion of the children due to changes in personal data (telephone number or place of residence) that occurred over a long period of time. A large proportion of parents of healthy early school-age children refused to come to their children’s evaluation. Due to difficulties conducting the study, we had to exclude patients with severe HIE, which reduced the sample size. Another limitation is that data on problems in learning were evaluated on the basis of the parents’ subjective opinion. We could not confirm the changes found in the ultrasound because, in 2007–2008, when the study was performed, brain MRI was not performed on newborns in Lithuania.

## 5. Conclusions

Our study showed that, in children who experienced perinatal asphyxia or hypoxia but did not have cerebral palsy and in cases where therapeutic hypothermia was not applied, cognitive assessment scores at an early school age were significantly lower than those in the group of healthy children, and were at a low average level. The results of our study will help parents become acquainted with possible remote outcomes at early school age, and help with the early initiation of adequate therapies to reduce poor neurological and neuromotor outcomes.

## Figures and Tables

**Table 1:** Children’s characteristics.

Characteristics	Case Group	Control Group
	*n* = 32	*n* = 16
**Sex, *n* (%)**		
boys	13 (40.6)	7 (43.8)
girls	19 (59.4)	9 (56.3)
**Age (years)**		
Min.	8.03	8.05
Max.	9.08	9.09
M(SD)	8.8 (0.417)	8.7 (0.45)
**Birth weight (g), *n* (%)**		
<3500	16 (50)	5 (31.25)
>3501	16 (50)	11 (68.75)
M(SD)	33.3 (10.05)	33.7 (8.12)
**Gestational age at birth (weeks), *n* (%)**		
37	2 (6.3)	1 (6.3)
38	3 (9.4)	0 (0)
39	8 (25.0)	5 (31.3)
40	13 (40.6)	5 (31.3)
41	6 (18.8)	5 (31.3)
M(SD)	39.56 (1.105)	39.81 (1.109)
**Apgar score at 1 min.**		
Min.	0	8
Max.	7	10
M (SD)	4.3 (1.7)	9.1 (0.6)
**Apgar score at 5 min.**		
Min.	4	9
Max.	9	10
M (SD)	6.94 (1.1)	9.5 (0.5)
**Extent of cardiopulmonary resuscitation after birth, (%)**		
No resuscitation measures applied	0 (0)	16 (100)
IS	3 (6.3)	0 (0)
IS-PPV	25 (52.1)	0 (0)
IS-PPV-CC	2 (4.2)	0 (0)
IS-PPV-CC-M	2 (4.2)	0 (0)
**HIE, *n* (%)**		
not detected	5 (15.6)	16 (100)
mild	15 (46.9)	0 (0)
moderate	12 (37.5)	0 (0)

M(SD)—mean (standard deviation); HIE—hypoxic-ischemic encephalopathy; IS—initial steps group; IS-PPV—initial steps and positive-pressure ventilation group; IS-PPV-CC—initial steps, positive-pressure ventilation and chest compressions group; IS-PPV-CC-M—initial steps, positive-pressure ventilation, chest compressions, and medications group.

**Table 2:** Qualitative Description of Composite Scores.

Composite Score	Classification	Theoretical Normal Curve
130 or above	Very superior	2.2
120–129	Superior	6.7
110–119	High average	16.1
90–109	Average	50.0
80–89	Low average	16.1
70–79	Borderline	6.7
69 or below	Extremely low	2.2

**Table 3:** Neurological examination, motor function, health-related quality-of-life, and cognitive assessments at an early school age.

Variable	Case Group (*n* = 32), Proc. (*n*)	Control Group (*n* = 16), Proc. (*n*)	*p* (χ^2^)
**Abnormalities in neurological examination**	43.8 (14)	25 (4)	0.21
Changes in cranial nerves	0.0 (0)	6.3 (1)	0.15
Changes in upper and lower limbs	40.6 (13)	12.5 (2)	0.05
Changes in cerebellar function	15.6 (5)	0.0 (0)	0.1
Gait disorders	9.4 (3)	0.0 (0)	0.2
Muscle tone disorders	9.4 (3)	0.0 (0)	0.2
Abnormalities detected during neurological examination (**tics, myoclonus, tremor, muscle atrophy**)	18.8 (6)	6.3 (1)	0.21
**Epilepsy**	12.5 (4)	0.0 (0)	0.14
**Neuromotor function (GMFCS)**			
No abnormality	75 (24)	87.5 (14)	0.55
Level I	21.9 (7)	12.5 (2)	
Level II	3.1 (1)	0.0 (0)	
Level III	0.0 (0)	0.0 (0)	
Level IV	0.0 (0)	0.0 (0)	
Level V	0.0 (0)	0.0 (0)	
**Health-related quality of life (HUI3)**			
***Vision***			
Level 1	87.5 (28)	87.5 (14)	1.0
Level 2	12.5 (4)	12.5 (2)	
Level 3	0.0 (0)	0.0 (0)	
Level 4	0.0 (0)	0.0 (0)	
Level 5	0.0 (0)	0.0 (0)	
Level 6	0.0 (0)	0.0 (0)	
***Hearing***			
Level 1	96.9 (31)	100 (16)	0.48
Level 2	3.1 (1)	0.0 (0)	
Level 3	0.0 (0)	0.0 (0)	
Level 4	0.0 (0)	0.0 (0)	
Level 5	0.0 (0)	0.0 (0)	
Level 6	0.0 (0)	0.0 (0)	
***Speech***			
Level 1	93.8 (30)	93.8 (15)	1.0
Level 2	6.3 (2)	6.3 (1)	
Level 3	0.0 (0)	0.0 (0)	
Level 4	0.0 (0)	0.0 (0)	
Level 5	0.0 (0)	0.0 (0)	
Level 6	0.0 (0)	0.0 (0)	
***Emotion***			
Level 1	100 (32)	100 (16)	1.0
Level 2	0.0 (0)	0.0 (0)	
Level 3	0.0 (0)	0.0 (0)	
Level 4	0.0 (0)	0.0 (0)	
Level 5	0.0 (0)	0.0 (0)	
Level 6	0.0 (0)	0.0 (0)	
***Pain***			
Level 1	90.6 (29)	81.3 (13)	0.65
Level 2	6.3 (2)	12.5 (2)	
Level 3	3,1 (1)	6.3 (1)	
Level 4	0.0 (0)	0.0 (0)	
Level 5	0.0 (0)	0.0 (0)	
Level 6	0.0 (0)	0.0 (0)	
***Ambulation***			
Level 1	75.0 (24)	93.8 (15)	0.12
Level 2	25.0 (8)	6.3 (1)	
Level 3	0.0 (0)	0.0 (0)	
Level 4	0.0 (0)	0.0 (0)	
Level 5	0.0 (0)	0.0 (0)	
Level 6	0.0 (0)	0.0 (0)	
***Dexterity***			
Level 1	93.8 (30)	100 (16)	0.59
Level 2	3.1 (1)	0.0 (0)	
Level 3	3.1(1)	0.0 (0)	
Level 4	0.0 (0)	0.0 (0)	
Level 5	0.0 (0)	0.0 (0)	
Level 6	0.0 (0)	0.0 (0)	
***Cognition***			
Level 1	68.8 (22)	87.5 (14)	0.36
Level 2	25.0 (8)	12.5 (2)	
Level 3	6.3 (2)	0.0 (0)	
Level 4	0.0 (0)	0.0 (0)	
Level 5	0.0 (0)	0.0 (0)	
Level 6	0.0 (0)	0.0 (0)	

χ^2^-Chi-squared criterion.

**Table 4:** Cognitive assessments at an early school age.

Cognitive Assessments	Case Group (*n* = 32) Mean (SD)	Control group (*n* = 16) Mean (SD)	*p*
Full IQ	87 (16.86)	107 (12.15)	<0.001
Verbal IQ	89 (17.45)	105 (11.55)	0.002
Verbal Comprehension Index	89 (17.36)	105 (10.74)	0.002
Working Memory Index	89 (15.68)	104 (11.84)	0.002
Performance IQ	87 (16.51)	108 (15.48)	<0.001
Perceptual Organization Index	85 (15.71)	105 (15.93)	<0.001

IQ—intelligence quotient. SD—standard deviation.

**Table 5:** IQ scores of 85 or lower at an early school age.

Cognitive Assessments	Case Group % (*n*)	Control Group % (*n*)	*p*
Full IQ	18.8 (6)	12.5 (2)	0.58
Verbal IQ	18.8 (6)	6.3 (1)	0.25
Verbal Comprehension Index	18.8 (6)	12.5 (2)	0.58
Working Memory Index	25.0 (8)	18.8 (3)	0.63
Performance IQ	28.1 (9)	12.5 (2)	0.23
Perceptual Organization Index	37.5 (12)	12.5 (2)	0.075

IQ—intelligence quotient.

**Table 6:** Mean IQ coefficient scores at an early school age in children with and without mental retardation at the age of 1 year.

IQ Coefficient Scores at an Early School Age	Slight Mental Retardation at the Age of 1 Year (*n* = 4) Mean (SD)	Normal Mental Development at the Age of 1 Year (*n* = 27/8 *) Mean (SD)	*p*
Full IQ	75.8 (21.1)	101.8 (15.1)/111.5 (21.1) *	0.027/0.017 *
Verbal IQ	78.0 (15.8)	102.3 (15.4)/110.1 (9.76) *	0.01/0.006 *
Verbal Comprehension Index	79.0 (13.3)	101.9 (15.5)/110.1 (9.70) *	0.018/0.006 *
Working Memory Index	76.5 (20.0)	100.3 (12.8)/107.4 (6.30) *	0.014/0.008 *
Performance IQ	77.3 (25.3)	100.7 (16.6)/110.9 (16.50) *	0.072/0.061 *
Perceptual Organization Index	77.5 (23.5)	97.4 (17.1)/105.0 (19.50) *	0.067/0.05 *

IQ—intelligence quotient; SD—standard deviation; * to specify for reliability, we randomly selected *n* = 8 from *n* = 27.

## Data Availability

Data is contained within the article.
